# Stem Cell Transplantation in the Treatment of Type 1 Diabetes Mellitus: From Insulin Replacement to Beta-Cell Replacement

**DOI:** 10.3389/fendo.2022.859638

**Published:** 2022-03-18

**Authors:** Xin-Xing Wan, Dan-Yi Zhang, Md. Asaduzzaman Khan, Sheng-Yuan Zheng, Xi-Min Hu, Qi Zhang, Rong-Hua Yang, Kun Xiong

**Affiliations:** ^1^ Department of Endocrinology, Third Xiangya Hospital, Central South University, Changsha, China; ^2^ Clinical Medicine Eight-Year Program, Xiangya School of Medicine, Central South University, Changsha, China; ^3^ The Research Centre for Preclinical Medicine, Southwest Medical University, Luzhou, China; ^4^ Department of Anatomy and Neurobiology, School of Basic Medical Science, Central South University, Changsha, China; ^5^ Department of Burn and Plastic Surgery, Guangzhou First People’s Hospital, School of Medicine, South China University of Technology, Guangzhou, China; ^6^ Hunan Key Laboratory of Ophthalmology, Changsha, China; ^7^ Key Laboratory of Emergency and Trauma, Ministry of Education, College of Emergency and Trauma, Hainan Medical University, Haikou, China

**Keywords:** type 1 diabetes mellitus, stem cell, β-cell, immunotolerance, transplantation

## Abstract

Type 1 diabetes mellitus (T1DM) is an autoimmune disease that attacks pancreatic β-cells, leading to the destruction of insulitis-related islet β-cells. Islet β-cell transplantation has been proven as a curative measure in T1DM. However, a logarithmic increase in the global population with diabetes, limited donor supply, and the need for lifelong immunosuppression restrict the widespread use of β-cell transplantation. Numerous therapeutic approaches have been taken to search for substitutes of β-cells, among which stem cell transplantation is one of the most promising alternatives. Stem cells have demonstrated the potential efficacy to treat T1DM by reconstitution of immunotolerance and preservation of islet β-cell function in recent research. cGMP-grade stem cell products have been used in human clinical trials, showing that stem cell transplantation has beneficial effects on T1DM, with no obvious adverse reactions. To better achieve remission of T1DM by stem cell transplantation, in this work, we explain the progression of stem cell transplantation such as mesenchymal stem cells (MSCs), human embryonic stem cells (hESCs), and bone marrow hematopoietic stem cells (BM-HSCs) to restore the immunotolerance and preserve the islet β-cell function of T1DM in recent years. This review article provides evidence of the clinical applications of stem cell therapy in the treatment of T1DM.

## 1 Introduction

Diabetes mellitus (DM) characterized by hyperglycemia, caused by insufficient insulin secretion or insulin resistance, is a group of chronic metabolic diseases. According to the International Diabetes Federation (IDF), the global adult diabetes population will exceed 537 million by 2021, and more than three-fourths of people with diabetes live in low- and middle-income countries, indicating that diabetes disproportionately affects the poor (http://www.diabetesatlas.org/). Diabetes is classified into four types: type 1 diabetes mellitus (T1DM), type 2 diabetes mellitus (T2DM), gestational diabetes mellitus (GDM), and monogenic diabetes mellitus (1–5). T1DM is an autoimmune disease, where autoreactive T cells attack pancreatic β-cells, leading to insulitis-related islet β-cell destruction, which results in an absolute lack of insulin secretion causing hyperglycemia, abnormal glucose metabolism, and lifelong dependence on exogenous insulin. The majority of T1DM patients have poor blood glucose control and large blood sugar fluctuation. Chronic hyperglycemia results in the development of serious complications associated with diabetes, such as microvascular and macrovascular complications, reducing the quality of life and causing a considerable economic burden on T1DM patients and the society (6). The incidence rate of T1DM is increasing every year around the world (7, 8). Although there is evidence that a combination of genetic susceptibility and environmental factors can increase the risk of immune disorder in T1DM patients, the exact etiology of the impaired immune system in T1DM is still unclear. More scientific efforts are needed to prevent β-cell loss and improve the quality of life in T1DM.

## 2 The Difficulties of Insulin Replacement and β-Cell Replacement in T1DM

At present, the treatment and preventive options for T1DM are limited, mainly through insulin replacement therapy. T1DM cannot be cured; patients must rely on exogenous insulin injections for the rest of their lives to maintain glycemic control. Lente and NPH insulin were the only effective methods for the treatment of T1DM in the past (9, 10). In recent years, novel approaches to insulin treatment, such as the introduction of glycosylated hemoglobin assays (HbA1c) and continuous glucose monitoring (CGM), have been used, and the effectiveness of basal/bolus therapy using portable continuous subcutaneous insulin infusion (CSII) pumps and closed-loop artificial pancreas system has been demonstrated. Artificial pancreas combining CGM with CSII pumps could automatically administer an appropriate insulin dose *via* a dosing algorithm. Some randomized controlled trials proved that the artificial pancreas system could efficiently adjust the glycemic index by automatically delivering exogenous insulin with dosing algorithms based on sensor glucose levels (11). However, the lag time of glycemia detected by CGM and the risk of hypoglycemia and infections limit the application of artificial pancreas, and some of the patients with unawareness of hypoglycemic events such as brittle type T1DM are not qualified to use the artificial pancreas (12, 13). Also, insulin replacement therapy can only supplement the missing insulin and cannot fundamentally restore the function of the pancreas. Although these achievements can better manage blood glucose and large blood sugar fluctuation in T1DM, they can hardly prevent the occurrence of a series of complications, including microvascular, macrovascular, and neuropathy complications (14, 15). As a result, many adjunctive therapies, such as dietary and weight management, nutrition therapy, physical activity and exercise, and some drugs used to treat T2DM, have been proposed to treat T1DM, which alleviate blood glucose fluctuation and reduce the lifetime risk of complications to some extent, but their effectiveness is limited. Therefore, it is very important to develop better technology and equipment for diagnosis and treatment options to prevent T1DM (16–18).

β-Cell replacement has also been proven as a curative measure in T1DM, which may be achieved through pancreas or islet transplantation in selective candidates (19). Pancreatic transplantation has the potential of re-establishing physiologic-regulated insulin production, obviously decreasing the risk of hypoglycemic unawareness and finally decreasing the longtime risk of mortality from severe hypoglycemic complications (20). Since 2000, β-cell replacement through intrahepatic isolated islet transplantation has proven efficacious, indicating that islet transplantation is also an important option in the treatment of T1DM (21). Compared with the artificial pancreas system, islet transplantation and pancreatic transplantation were the better options to relieve the symptom of T1DM patients with unawareness of hypoglycemic events such as brittle type T1DM for a long time (22). T1DM patients can be clinically alleviated through improved control of the levels of blood glucose and restored awareness of hypoglycemia, resulting in the prevention of several life-threatening complications associated with diabetes, such as diabetic foot, microvascular and macrovascular diseases, kidney failure, nerve damage, and blindness (23). During the process of pancreatic or islet transplantation, both the autoimmune and alloimmune systems are still major threats to increase the transplantation risk. Patients treated with cell replacement therapies require immunosuppressive drugs as life-long treatment, and in many cases, these drugs lead to toxicities and side effects that made the adoption of this treatment strategy limited to only the most severe disease cases, inhibiting the widespread adoption of pancreatic or islet transplantation therapies in T1DM (24).

Besides the immune problem, the logarithmic increase in the global population of people with diabetes, the limited donor supply, and the need for lifelong immunosuppression restrict its widespread use (25). Numerous therapeutic approaches have been reported to solve this problem, including the search for β-cell substitutes, porcine islet xenotransplantation, and stem cell transplantations, which present solutions to the donor shortage and may be the most likely alternatives (26, 27).

Although the artificial pancreas system and pancreatic transplantation in T1DM can normalize and improve glycemic control in T1DM, the application of artificial pancreas systems and pancreatic transplantation is still limited due to their shortage. To solve the problem, stem cell transplant is a promising new strategy for patients with T1DM. There are many advantages of stem cells in the treatment of T1DM: first of all, stem cells such as bone marrow-derived stem cells (MSCs) can easily be obtained from bone marrow, umbilical cord blood, adipose tissue, etc. compared with islet and pancreas; secondly, the pluripotent stem cells could differentiate into β-cells and increase the secretion of insulin; thirdly, stem cells can moderate the immunome effect by inhibiting T-cell proliferation and reduce the inflammatory response, which can protect β-cells from autoimmune attack; and finally, stem cells can secrete cytokines by paracrine effects to enhance the antioxidant and proliferation ability of cells, which can help improve the survival of β-cells. To better understand the constitution of immunotolerance and preservation of islet β-cell function, we reviewed the progression of stem cells in recent years and tried to provide support for the clinical applications of stem cell therapy in the treatment of T1DM, especially in the brittle type T1DM.

## 3 Stem Cell Transplantation Therapy for T1DM

**Figure 1:**
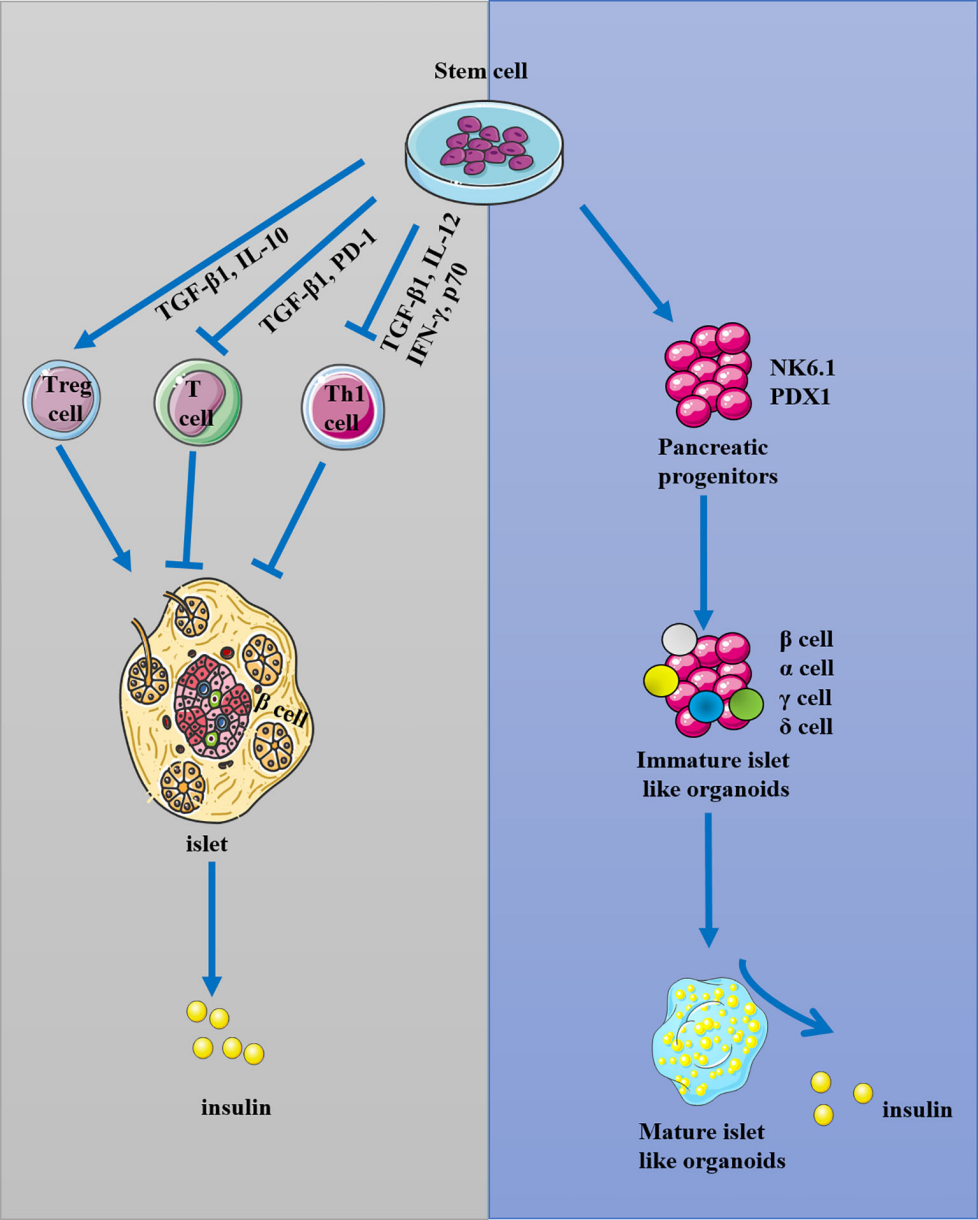
The possible mechanism of stem cells in the treatment of T1DM. Stem cells were used for the reconstitution of immunotolerance through activating T regulatory cells (Treg) and inhibiting T and Th1 cells, and they could also be used for the preservation and regeneration of β-cells.

Stem cells are undifferentiated cells capable of self-renewal, giving rise to virtually any tissue or organ (28–33). Stem cells can be grouped into four broad categories based on their origin: adult stem cells (ASCs), fetal stem cells (FSCs), embryonic stem cells (ESCs), and induced pluripotent stem cells (iPSCs). iPSCs and ESCs are pluripotent stem cells (PSCs), whereas ASCs are unipotent or oligopotent (34–36). PSCs, such as human-induced PSCs (iPSCs) and human embryonic stem cells (ESCs), offer a reproducible source of human cells at a very early developmental stage with the potential to form any cell type in the adult body (37–39). iPSCs, human cord blood-derived multipotent stem cells (CB-SCs), hematopoietic stem cells (HSCs), and MSCs were used for the preservation of β-cells by islet protection and regeneration, and another potent function of stem cells is the ability to re-establish peripheral tolerance toward β-cells through remodeling of the immune response as well as through inhibition of autoreactive T-cell function (40, 41). In general, stem cells can increase the mass of islets by the ability of differentiation to β-cells-like organoids, and reconstitute immunotolerance by inhibiting the immune response of T cell and Th1 cells through TGF-β and inflammatory pathways (**Figure 1**). As T1DM is featured as an autoimmune disease by activating immune cells to attack and destroy pancreatic β-cells, the immunomodulatory properties of stems cells and its potential ability of differentiation into insulin-producing cells should be considered when using stem cell therapy for T1DM treatment.

### 3.1 MSC Transplantation in T1DM

MSCs are one of the best candidate cells used as cell therapy for T1DM. MSCs are fibroblast-like, multipotent stromal, non-hematopoietic cells that could easily be sourced from various tissues, including adipose tissue, bone marrow, and umbilical cord blood (42). MSCs rapidly undergo mesodermal lineage differentiation, such as adipocytes, myoblasts, cardiomyocytes, chondrocytes, and β-cell-like cells (43–45). The bone marrow and umbilical cord blood could be separated over a gradient of Percoll by density gradient centrifugation to collect the MNCs, and the MNCs were washed with PBS and transferred to a 100-mm culture dish to induce MSCs. The redundant tissues such as arteries and veins were removed from the adipose tissue, human umbilical cord, etc. The region of the remaining adipose or human umbilical cord tissues was diced into small fragments and seeded into a 100-mm culture dish to collect the MSCs. The induced MSCs were stored in liquid nitrogen and cultured for up to five passages for transplantation *via* intravenous injection. The characteristics of MSCs were defined by the International Society for Cell Therapy (ISCT) as follows: adherence to plastic; expression of the surface molecules CD73, CD90, and CD105 in the absence of CD34, CD45, HLA-DR, and CD14 or CD11b and CD79a or CD19; and the capacity for differentiation to adipocytes, osteoblasts, and chondroblasts *in vitro* (46). The potential of MSCs as a cell-based therapy in the treatment of immunologic disorders has been well established (47). MSCs can alter the microenvironment in tissues and promote existing β-cell survival and regeneration, resulting in increased β-cell mass and normal blood glucose recovery (48–51). Injection of bone marrow MSCs into diabetic mice can increase insulin levels and downregulate hyperglycemia; the exosomes derived from human umbilical cord stem cells (hUCMSCs) can enhance insulin sensitivity (52). Similarly, monotherapy with human umbilical cord MSCs reverses autoimmunity, promotes islet cell regeneration, and improves blood glucose control (53–55).

The allogeneic MSCs have been attempted in clinical trials, which can improve the level of insulin and C-peptide and reduce blood glucose. Although MSC xenotransplantation was not used in the clinics, several pieces of evidence showed that human-derived MSCs could alleviate the diabetic symptom through β-cell-like organoid differentiation and immunomodulation in NOD mice, rats, and monkeys, while far-red light, gene editing, and other modifications could enhance the function of MSCs in a T1DM animal model, indicating that the intervening and xenograft MSCs are the potential option for T1DM treatment.

#### 3.1.1 Immunomodulatory Ability of MSCs

MSCs can protect β-cell, increase the secretion of insulin, and reduce glycemia in patients with T1DM by regulating the immune system. The application of MSCs in eliminating autoimmune diseases has been fully proven in an animal model, and MSCs have a wide range of regulatory effects on immune cells. Domouky et al. showed that MSCs could reduce hyperglycemia in diabetic rats on day 15 (56). The inhibition of T-cell proliferation in islets and the presence of increased Treg in T1DM were features of MSCs’ autoimmune properties (57). Shigemoto-Kuroda et al. developed T1DM mouse models for autoimmune diseases and discovered that MSCs could suppress type 1 helper T cell (Th1) development and delay the onset of T1DM in mice. CD4^+^ cells were found in significant numbers in the islets of mice treated with PBS, while fewer CD4^+^ cells were found in the islets of MSC-treated mice. The level of insulin in plasma was increased by MSC treatment, and there was a significant reduction in the production of IL-12, IFN-γ, p70, and tumor necrosis factor (TNF) (58). Bassi et al. isolated MSCs from epididymal fat tissue from 8-week-old male Balb/c mice and characterized through immunophenotyping its capacity to prevent the proliferation of CD4^+^ T cells (59, 60). Treatment of NOD mice with MSCs attenuated hyperglycemia of early-onset autoimmune diabetes and increased amylin levels, reflective of autoimmune diabetes improvement; reduced the amount of inflammatory cell infiltration, maintaining insulin expression in pancreatic islets by suppressing the Th1 immune response in the pancreas; and promoted the high expression of active TGF-β1 (60). Meanwhile, syngeneic MSCs were detected for a significantly longer period, albeit with diminishing persistence in immune-deficient mice model (61). In another study, van Megen et al. found that activation of MSCs can take up and process antigen and increase HLA-DR expression and immune inhibitory markers, while their metabolic profile was maintained without enhancing T-cell proliferation. MSCs can also enhance immunosuppressive capacity without stimulating alloreactive T cells (62). In an *in-vitro* study, Montanucci et al. provided preliminary evidence that immunoisolatory microcapsule-hHUCMS (CpS-hUCMS) may represent a functional biohybrid artificial system, where molecular products can induce effective immunomodulatory effects *in vitro* and in T1DM patients, making it possible to further clarify their therapeutic potential in humans (63). Montanucci et al. isolated and microencapsulated human umbilical cord Wharton jelly-derived mesenchymal stem cells (hUCMS) for xenograft (TX) in a spontaneous T1DM mouse model (NOD mice). At 10 days of TX, Treg cells did not increase, while at 216 days of TX, CD4^+^ CD25 high cells increased in terms of both percentage and number. Further research found that at 216 days of TX, only the mild T1DM NOD mice presented sustained and full alleviation of hyperglycemia, while no alleviation of hyperglycemia was observed in severe T1DM NODs. These findings suggested that the successful hUCMS therapy approach for the treatment of T1DM in NOD mice depended on the stage of the T1DM disease process, with severe T1DM NODs exhibiting a continuous decrease in residual β-cell mass (64). All these results provide encouraging first steps in the clinical translation of the use of preactivated MSCs as a cellular immune intervention therapy, which helps to treat inflammatory and autoimmune disorders, including T1DM.

#### 3.1.2 Islet Protection and Regeneration Ability of MSCs

MSCs can increase the mass of islets and the survival of β-cells by differentiation to β-cell-like cells. MSCs rapidly undergo mesodermal lineage differentiation to β-cell-like cells, and the transdifferentiation of MSCs into insulin-producing cells was successfully attempted *in vitro*, with the pancreatic and duodenal homeobox 1 (PDX-1), the key marker that was present in the transdifferentiation of MSCs to insulin-producing cells (65). Chen et al. successfully differentiated MSCs into pancreatic islet β-cell-like cells by pre-inducing in L-DMEM with 10 mmol/L nicotinamide and 1 mmol/L β-mercaptoethanol for 24 h and re-inducing in serum-free H-DMEM with 10 mmol/L nicotinamide and 1 mmol/L mercaptoethanol for another 10 h. These induced islet β-cell-like cells with similar morphological characters to pancreatic islet cells and promoted the transcription, translation, and excretion of insulin, which could effectively control the level of blood glucose in diabetic rats (66). Similar results were reported by several other groups that islet-like clusters can be formatted *in vitro* by cultured MSCs given the appropriate procedure (67, 68). Human umbilical cord Wharton jelly cells (hUCWJCs) are a subtype of MSCs, which were transplanted into a T1DM mouse model with renal damage, and the therapeutic effect of transplantation was evaluated. It was found that hUCWJCs can promote the level of C-peptide and insulin in mice, which certified the potential of intraperitoneal injection of hUCWJCs and the ability of hUCWJCs to migrate to damaged tissues to enhance the secretion of insulin from non-pancreatic local cells (69).

#### 3.1.3 MSC Transplantation in Clinical Trials

MSCs have been used in human clinical trials, showing that stem cell transplantation has beneficial effects on T1DM. In an open-label, non-randomized, parallel-armed prospective study, Lu et al. enrolled 53 participants including 33 adult-onset (≥18 years) and 20 juvenile-onset T1DM (ChiCTR2100045434). The results revealed that an intravenous dose of allogeneic UC-MSCs was safe in people with newly diagnosed T1DM at 12 months of follow-up, which probably led to better islet β-cell protection compared with standard treatment alone during the first year after diagnosis (70). Cai et al. proved that transplantation of UC-MSCs was safe and associated with moderate improvement of metabolic measures in patients with established T1DM too (NCT01374854) (50). Another clinical trial revealed that MSC injection through liver puncture could successfully decrease the levels of insulin, islet cells, and glutamic acid decarboxylase (GAD) antibody in two patients within 1 year, with a decreased concentration of blood glucose and HbA1c and increased concentration of C-peptide, indicating immune regulatory cell tolerance (71).

### 3.2 HSC Transplantation in T1DM

The conception of HSCs was generated in the 1950s with the discovery that intravenously injected bone marrow cells could rescue irradiated mice from lethality through re-establishing blood cell production (72). The ability to manage expansion and the characteristics of self-renewal of the hematopoietic compartment while maintaining the capacity for differentiation into HSCs were demonstrated (72, 73). Peripheral hematopoietic stem cells are mobilized with cyclophosphamide and granulocyte colony-stimulating factors. Leukapheresis using a continuous-flow blood cell separator was initiated when the rebounding CD34^+^ cells reached 10 cells/μl. Apheresis was continued daily until the number of harvested progenitor cells reached a minimum of 3.0 × 10^6^ CD34^+^ cells/kg body weight. Unmanipulated peripheral blood stem cells were frozen in 10% dimethyl sulfoxide in a rate-controlled freezer and stored in the vapor phase of liquid nitrogen (74). Then, the collected cells were injected intravenously. HSCs have proven to be safe in human subjects and have been widely utilized as an effective treatment for hematological malignancies (75). Recently, HSCs have been used in T1DM for the suppressed function of the immune system response in both *in-vitro* and *in-vivo* studies.

#### 3.2.1 Immunomodulatory Ability of HSCs

Immunomodulatory activity is the most important ability of HSCs in patients with T1DM. HSCs can inhibit the occurrence of T1DM (76–78). Patients with recent-onset T1DM have been triumphantly reverted to euglycemia by autologous hematopoietic stem and progenitor cell transplantation (AHSCT), and modulation of autologous hematopoietic stem and progenitor cells (HSPCs) with prostaglandins (PGs) *in vitro* enhances their immunoregulatory properties through increasing the expression of the immune checkpoint-signaling molecule PD-L1 (79). Wang et al. demonstrated a lower proportion of proliferating T conventional cells (Tcon) and a higher absolute number and percentage of Treg cells in pancreatic lymph nodes from resistant mice among the younger recipients compared to the rapid progressors among the older recipients, and older NOD mice progressed more rapidly to the end stage of diabetes (80). Although mixed chimerism with MHC-matched non-autoimmune donor bone marrow (BM) transplants did not prevent T1DM in NOD mice models, induction of either mixed or complete chimerism with MHC-mismatched BM transplants inhibited T1DM in the same mice (81). This limited the translational applications of HSCs to reshape the autoimmune response by myeloablative agents/approaches. The genetically modified HSCs were used to overcome the disadvantage. *Ex-vivo* genetic manipulation of NOD HSCs to encode proinsulin and transgenically target MHC class II could successfully prevent T1DM onset (78, 82). The increased CXCL12 (SDF-1) level in bone marrow-derived HSCs of NOD mice is considered to change the transport of HSCs and peripheral dendritic cells, which is conducive to the occurrence of T1DM (78, 83).

#### 3.2.2 HSC Transplantation in Clinical Trials

D’Addio et al. enrolled 65 individuals with newly diagnosed T1DM in three independent clinical trials, where the patients transplanted with HSCs showed enhanced C-peptide levels at 6 months after treatment compared with baseline, and the immune system showed an overall stabilization in the remaining follow-up period (84). Gu et al. performed a parallel-assignment, phase-II prospective, non-randomized trial, in which 20 patients were treated only with insulin injections and 20 received autologous hematopoietic stem cell therapy (AHSCT). The results demonstrated the beneficial effects of AHSCT in patients with recent-onset T1DM by increasing the concentration of C-peptide and inducing insulin independence, and the safety and good tolerability of AHSCT compared with conventional intensive insulin therapy was also certified (77). Another clinical trial in Ning’s research also proved that AHSCT was safe without a reduction in the diversity of T-cell receptor (TCR) repertoires, and TCR repertoires tended to be more stable after AHSCT (85). The clinical trial data also showed significant direct correlations between HSPC levels and the coefficient of variation of glucose levels or time in hypoglycemia, which were weaker in patients with long-standing diabetes than in those with short-term diabetes (86). HSC transplantation improves glycated hemoglobin levels in a time-dependent manner (87).

### 3.3 ESC and iPSC Transplantation in T1DM

ESCs and iPSCs are PSCs that can regenerate the islet β-cells and immune cells through differentiating, which helps to increase the mass of β-cells. D’Amour et al. firstly proved that ESC-derived β-cells could be successfully generated through the *in-vitro* recapitulation of pancreatic islets and β-cell physiological development by stepwise application of specific factors (88, 89). iPSCs, reprogrammed from somatic cells, have a similar ability to differentiate and proliferate like ESCs. iPSCs collected from the umbilical cord at birth have the potential for self-renewable multipotency and can differentiate into various lineages such as islets (31, 59, 90). Hence, iPSCs provide a promising platform to produce insulin-secreting cells *in vitro*. However, the utilization of ESCs and iPSCs is less due to law restrictions in many countries, so there is little clinical research on ESCs and iPSCs.

#### 3.3.1 Islet Protection and Regeneration Ability of ESCs and iPSCs

Transplantation of ESCs or iPSCs in T1DM can regenerate the islet β-cells and increase β-cell mass through differentiating to insulin-producing cells (IPCs), pancreatic progenitors, islet organoids, and interspecific pancreatic chimeras, which benefited the treatment of T1DM. Rezania et al. cultured iPSCs expressing key markers of mature β-cells such as insulin *in vitro* and obtained cells which have functional similarities to human islets; the iPSCs rapidly reversed hyperglycemia in streptozotocin (STZ)-induced diabetic mice through increasing the level of insulin and C-peptide when transplanted *in vivo* (91). iPSCs can be generated from the skin fibroblasts of T1DM patients. These iPSCs can differentiate into pancreatic cell lineages and generate T1DM SC β-cells, making autologous stem cell-derived pancreatic progeny transplantation for T1DM possible (92). Korytnikov and Nostro isolated hPSCs successfully in a lab and then transplanted them to mice models to monitor their developmental potential *in vivo*, and they found that mice transplanted with multipotent pancreatic progenitor cells can form all pancreatic lineages *in vivo* (93). Nadav et al. used single-cell RNA sequencing of differentiating β-cells and revealed that ESC differentiation toward the mature β-cell phenotype can be tracked at each stage through monitoring the expression of markers identifying each intermediate progenitor, such as the β-cell marker insulin, endocrine precursor marker neurogenin 3, NK6 homeobox (NKX6.1), and PDX-1 (89, 94). They also proved that WNT inhibition and bone morphogenetic protein (BMP) activation could modulate the ratio of progenitors and endocrine cells, which shed light on a possible gene editing target for ESC differentiation toward the mature β-cells (89).

#### 3.3.2 Immunomodulatory Ability of ESCs and iPSCs

Haque et al. characterized autoantigen-specific naturally occurring Treg-like iPSC-Tregs and proved that adoptive transfer of ovalbumin (OVA)-specific iPSC-Tregs greatly suppressed autoimmunity in the mouse model preventing the β-cells from destruction. These tissue-associated Tregs can effectively inhibit the migration and activity of the pathogenic immune cells and accumulate in the diabetic pancreas causing T1DM by downregulating the production of proinflammatory cytokine IFN-γ and suppressing the expression of ICAM-1 (95). Another report declared that pancreatic endoderm derived from hESCs can generate functional insulin-producing cells *in vivo* regardless of the presence of innate lymphoid cell elements, and the combination of CTLA4Ig and anti-CD40L mAbs can block hESC-PE graft rejection in immunocompetent mice, while regulatory T cells were not needed for the tolerance during hESC-PE transplantation (96).

To increase the function of stem cells, it is very important to sustain the regeneration and differentiation ability of stem cells and prevent the programmed death of stem cells *in vivo* (30, 97). Several approaches and conditions, including far-red light, genetic engineering, biological material scaffolds, nanofiber tubular, combination treatment with insulin or other drugs, microcapsules, and co-transplantation with more than one type of stem cells, were utilized to promote the survival, differentiation, and immunomodulatory ability of stem cells *in vivo* and *in vitro*. These preclinical attempts tried to derive pancreas islet cells, increase the number and function of Tregs, ameliorate the function of islets, and prevent β-cells more effectively (**Table 1**). Also, stem cells have been used in human clinical trials, which showed that stem cell transplantation had beneficial effects on T1DM, with no obvious adverse reactions (**Table 2**). Recently, an allogeneic, gene-edited, immune-evasive, stem cell-derived therapy for the treatment of T1DM was approved in Canada for clinical trial application (CTA) (CRISPR Therapeutics and ViaCyte, Inc. to start clinical trial of the first gene-edited cell replacement therapy for the treatment of T1DM, retrieved on November 16, 2021). This CRISPR therapeutics offered novel β-cell replacement therapies to address unmet T1DM needs. All these efforts are aimed at better promoting the effectiveness of stem cells, which proved to be a more viable option for the treatment of T1DM, lessening the suffering of the patients.

**Table 1:** Intervention strategies to improve the treatment effect of stem cells.

Type	Donor	Recipient	Pretreatment condition	Doses	Effect	Pathway/target/mechanism	Year/reference
hESCs	Human	Mice	Aggregates of hESC-derived PP with rat-derived MV in hydrogels	N/A	Accelerate the normalization of glycemia and persist long-term and promote beta-cell maturation	Improve the survival of PP cells by reducing hypoxia and apoptosis	2021 (98)
hMSCs	Human	Mice	Far-red light-activated human islet-like designer	N/A	Improve glucose tolerance, sustain blood glucose control, and attenuate both oxidative stress and development of multiple diabetes-related complications in the kidneys	Sustain fine-tuned secretion of insulin	2021 (99)
hPSCs	Human	*In vitro*	PD-L1 overexpression and HLA class I knockout by genome engineering	N/A	Provide protection from diabetes-specific immune recognition	Abrogate human diabetogenic CD8 T cells in activation	2021 (100)
hPSCs	Human	*In vitro*	Superporous agarose scaffolds	N/A	Enhance the function of the bioartificial pancreas	Sustain insulin production	2021 (101)
hESCs	Human	Mice/dog	Nanofibrous encapsulation	2,500 cluster cells/mouse	Corrected glucose levels immediately (within a week)	Sustain the survival of human SC-β cells in immunodeficient mice and immunocompetent mice and dogs	2021 (102)
hBM-cMSCs	Human	Rhesus monkey	Combined therapy with liraglutide	1.5 × 10^6^/kg	Reduce FBG and HbA1c	Immunomodulation by increased Tregs, IL-4, IL-10, and TGF-β1 and decreased IL-6 and IL-1β	2021 (103)
ADMSCs	Human	Mice	Co-culture with MIN6 cells	0.5 × 10^6^/mouse	Provide protection to MIN6 cells from streptozotocin	AKT and ERK pathway	2021 (43)
MSCs	Human	Mice	Generated human alpha-1 antitrypsin-engineered MSCs	N/A	Increased self-renewal, better migration, and multilineage differentiation abilities	Upregulate the expression of WNT3A, KDR, ICAM 1, VICAM1, MMP2, and IGF1	2021 (104)
hUCMS and hIDCs	Human	Mice	Co-microencapsulation of hUCMS and hIDC in AG	1 × 10^6^ hIDC + 1 × 10^6^ hUCMS/1.3 ml AG	Reverse the recent onset hyperglycemia in mice	Couple the immunoregulatory activities of hUCMS and insulin production by hIDC	2020 (105)
MSCs	Human	Rats	Combined therapy with insulin	2 × 10⁶ MSCs/kg	Decrease blood glucose level	Regulate the expression of leptin receptor, neuropeptide Y, and melanocortin-4 receptor	2020 (106)
ADMSCs	Human	Mice	Co-transplant with neonatal porcine islets	1 × 10^6^ MSCs/mouse	Improve glucose tolerance and stimulate serum porcine insulin	Secrete anti-inflammatory and proangiogenic factors	2019 (107)
MSCs	Mice	Mice Allograft	Intraperitoneal injection MSCs and MSC-conditioned medium together	1 × 10^6^ MSCs/mouse	More effective in induction of immunosuppressive effects	Reduce the inflammatory cytokines and increase inflammatory cytokine	2020 (108)
ESCs	Mice	Mice Allograft	MHC-mismatched	N/A	Prevent insulitis and T1D development	Increase the number and function of Tregs	2019 (109)
HSPCs	Human	Murine	Overexpression PD-L1 by genome engineering	1 × 10^6^/mouse	Inhibit the autoimmune response and revert diabetes	PD-L1/PD-1 pathway	2017 (110)
hESCs	Human	Mice	Alginate microencapsulation with CXCL12	1 × 10^6^/mouse	Enhance insulin secretion and enhance immunoisolation	CXCL12 pathway	2019 (111)
HSPCs	Human	Human Autograft	Modulation with prostaglandins	1 × 10^5^/mouse	Abrogate the autoreactive T-cell response	PD-L1 pathway	2018 (79)
MSCs	Mice	Mice Allograft	Co-transplantation with immature dendritic cells	2 × 10^5^/mouse	Decrease blood glucose and glycosylated hemoglobin levels	Inhibit the proliferation of T lymphocytes to induce immune tolerance	2017 (112)
iPSCs	Human	Mice	Transient demethylation treatment	N/A	Significantly improve the yield of PDX-1^+^ and NKX6.1^+^ cells	Rescue and generate the islet-like compact cell clusters	2017 (113)
MSCs	Mice	Mice Allograft	Overexpress TGF-β by genome engineering	5 × 10^5^/mouse	Improve insulin levels and suppress adverse immune responses	IFN-γ/IL-4 pathway	2017 (114)
hESCs	Human	*In vitro*	Barium alginate capsules	N/A	Increase cell proliferation and pancreatic differentiation.	TGF-β signaling	2016 (115)
MSCs	Rats	*In vitro*	Co-encapsulation within GLP-1 ligand-functionalized polyethylene glycol hydrogel	N/A	Improve islet function and stimulate cell survival	GLP-1 promotes the stimulation of insulin gene transcription, islet growth, and neogenesis	2017 (116)
ADMSCs	Mice	Mice Allograft	Intrasplenic injection or intrapancreatic injection	1 × 10^6^/mouse	Intrasplenic administration improves β-cell mass and insulin production	Increase pancreatic TGF-β levels	2015 (117)

hESCs, human embryonic stem cells; PP, pancreatic progenitors; hMSCs, human mesenchymal stem cells; hPSCs, human pluripotent stems; hBM-cMSCs, human clonal mesenchymal stem cells; FBG, fasting blood glucose; ADMSCs, adipose tissue-derived MSCs; N/A, not applicable; MSCs, mesenchymal stem cells; WNT3A, Wnt family member 3A; KDR, kinase insert domain receptor; ICAM-1, intercellular adhesion molecule 1; VICAM-1, vascular cell adhesion protein 1; MMP2, matrix metalloproteinase-2; IGF-1, insulin-like growth factor; iPSCs, induced pluripotent stem cells; hUCMS, human umbilical cord-derived mesenchymal stem cells; hIDCs, pancreatic islet-derived insulin-producing cells; AG, sodium alginate; HSPCs, hematopoietic stem and progenitor cells; PD-L1, programmed death ligand 1.

**Table 2:** Stem cell treatment of T1DM in human clinical trials.

Type	Case	Transplantation method	Observation	Effect	Side effect	Number of clinical trials	Year/reference
MSCs	27 (MSC-treated); 26 (control)	The MSC-treated group received repeat transplantation with an interval of 3 months, and each time for 1.0 × 10^6^ cells/kg was given.	Participants were followed up at 3, 6, and 12 months and yearly afterward	HbA1c levels decreased and C-peptide was significantly increased in the MSC-treated group	Had mild fever after MSC infusion	ChiCTR2100045434	2021 (70)
ASCs	8 (ASC treated); 5 (control)	1 × 10^6^ cells/kg ASCs and cholecalciferol (Vit D) 2,000 IU/day for 3 months	Participants were followed up at 3, 6, and 12 months and yearly afterward	Stability of C-peptide, better glucose control, and lower insulin requirement	Transient headache (*n* = 8), mild local infusion reactions (*n* = 7), tachycardia (*n* = 4), and abdominal cramps (*n* = 1)	NCT03920397	2020 (118)
AHSCT	20 (AHSCT); 20 (control)	HSCs were mobilized and collected from peripheral blood by leukapheresis and cryopreserved. Cells were injected intravenously after conditioning with CTX (200 mg/kg) and rabbit ATG (4.5 mg/kg)	Participants were followed up at 3, 6, 12, 18, 24, 36, and 48 months	Reduction of insulin dosage, decreased HbA1c and increased Cmax and area under the curve for C-peptide (AUCC)	Febrile neutropenia (*n* = 9), nausea and vomiting (*n* = 11), alopecia (*n* = 19), and blood component transfusions due to bone marrow suppression (*n* = 5)	NCT00807651	2018 (77)
CB-SCs	15	CB-SC-treated mononuclear cells (interaction for 2~3 h) were returned to the patient’s blood circulation *via* a dorsal vein in the hand with physiological saline; after 3 months, subjects received a similar second treatment	Follow-up visits were scheduled 2, 8, 12, 18, 26, 40, and 56 weeks after treatment for clinical assessments and laboratory tests	Restored the regeneration of naive CD^4+^ T cells and the function of β-cell in patients with residual β-cell function was rescued and without a significant linear decline	N/A	NCT01350219	2015 (119)
UC-MSCs	21 (UC-MSCs); 21 (control)	The dorsal pancreatic artery or its substitute was identified, and 60~80 ml BM-MNCs (106.8 × 10^6^/kg) plus 30~50 ml UC-MSCs (1.1 × 10^6^/kg) were sequentially infused within 30 min	N/A	HbA1c levels decreased, FBG decreased, and insulin dose requirements reduced	N/A	NCT01374854	2016 (50)
CB-SCs	12 (with CB-SCs educated); 3 (without CB-SCs educated)	The collected lymphocytes were transferred into the device for exposure to allogeneic CB-SCs for 2~3 h, then were returned to the patient’s circulation *via* a dorsal vein in the hand under gravity flow control (2 to 3 ml/min) with physiological saline	Follow-up visits were scheduled 4, 12, 24, and 40 weeks after treatment for clinical assessments and laboratory tests	Improved fasting C-peptide levels, reduced daily dose of insulin, increased Tregs, and reduced HbA1c	N/A	NCT01350219	2012 (120)
ASCs	7 (ASCs + VIT D); 4 (VIT D); 6 (control)	Allogenic ASC (1 × 10^6^ cells/kg) and cholecalciferol 2,000 UI/day for 6 months	Participants were followed up at T0 and after 1, 3, and 6 months	Improved fasting C-peptide levels and reduced HbA1c level	Four patients developed local thrombophlebitis within the first week and two had transient mild eye floaters during infusion, with no subsequent visual abnormalities. One patient developed central retinal vein occlusion at T3, with complete resolution at T6	NCT03920397	2021 (121)

T1DM, type 1 diabetes mellitus; BMI, body mass index; SCs, stem cells; MSCs, mesenchymal stem cells; hESCs, human embryonic stem cells; ASCs, adipose stem cells; HSCs, hematopoietic stem cells; BM-MSCs, bone marrow-derived mesenchymal stem cells; HbA1c, glycosylated hemoglobin assays; FBG, fasting blood glucose; Treg, T regulatory; hUCMS, human umbilical cord matrix stem cells; sBCs, stem cell-derived pancreatic beta-like cells; ADMSCs, adipose tissue-derived MSCs; CB-SCs, human cord blood-derived multipotent stem cells; UC-MSCs, umbilical cord mesenchymal stromal cells; VIT D, vitamin D; N/A, not applicable.

## 4 Concluding Remarks

In recent research, stem cell therapy has demonstrated itself as a rapidly expanding and potentially limitless source of β-cells to arrive at a cure for T1DM by reconstitution of immunotolerance and differentiation into islet β-cell clusters. As the immunosuppression affected the effect of transplantation of stem cells, stem cell intervention before transplantation could help preserve β-cells and remodel the immune response. However, several challenges, such as the ethical problem of autologous and allogeneic stem cells used to preserve the function of β-cells, still need resolution. Although research into β-cell replacement derived from stem cells is increasing every year, we must make more efforts in the future on the intervention with stem cell transplantation, which can help achieve remission of T1DM by β-cell replacement.

## Author Contributions

X-XW reviewed the literature, wrote the manuscript, and created the descriptive figures. X-MH and QZ edited the tables and figures. D-YZ and S-YZ assisted in the literature review. MK edited the manuscript. KX and R-HY revised the manuscript. All authors read and approved the final manuscript.

## Funding

This work was supported by the National Natural Science Foundation of China (81971891, 82172196, and 81772134), Key Laboratory of Emergency and Trauma (Hainan Medical University) of the Ministry of Education (KLET-202108), Hunan Province Natural Science Foundation of China (2018JJ3804), and the College Students’ Innovation and Entrepreneurship Project (S20210026020013).

## Conflict of Interest

The authors declare that the research was conducted in the absence of any commercial or financial relationships that could be construed as a potential conflict of interest.

## Publisher’s Note

All claims expressed in this article are solely those of the authors and do not necessarily represent those of their affiliated organizations, or those of the publisher, the editors and the reviewers. Any product that may be evaluated in this article, or claim that may be made by its manufacturer, is not guaranteed or endorsed by the publisher.
